# Oxidation of K^+^ Channels in Aging and Neurodegeneration

**DOI:** 10.14336/AD.2015.0901

**Published:** 2016-03-15

**Authors:** Federico Sesti

**Affiliations:** Department of Neuroscience and Cell Biology, Robert Wood Johnson Medical School, Rutgers University, Piscataway, NJ 08854, USA

**Keywords:** K+ channel, aging, ROS, neuron, endothelial cells, smooth muscle, neurodegenerative disease

## Abstract

Reversible regulation of proteins by reactive oxygen species (ROS) is an important mechanism of neuronal plasticity. In particular, ROS have been shown to act as modulatory molecules of ion channels—which are key to neuronal excitability—in several physiological processes. However ROS are also fundamental contributors to aging vulnerability. When the level of excess ROS increases in the cell during aging, DNA is damaged, proteins are oxidized, lipids are degraded and more ROS are produced, all culminating in significant cell injury. From this arose the idea that oxidation of ion channels by ROS is one of the culprits for neuronal aging. Aging-dependent oxidative modification of voltage-gated potassium (K^+^) channels was initially demonstrated in the nematode *Caenorhabditis elegans* and more recently in the mammalian brain. Specifically, oxidation of the delayed rectifier KCNB1 (Kv2.1) and of Ca^2+^- and voltage sensitive K^+^ channels have been established suggesting that their redox sensitivity contributes to altered excitability, progression of healthy aging and of neurodegenerative disease. Here I discuss the implications that oxidation of K^+^ channels by ROS may have for normal aging, as well as for neurodegenerative disease.

ROS are highly reactive molecules that are formed as a natural by-product of the metabolism of oxygen. ROS can react with proteins, DNA, cell membranes and other molecules as well as generate more reactive radicals [1]. As aging advances, the redox environment of the cell becomes altered in favor of oxidation by an increased production of ROS and/or a decrease in antioxidant defenses. Unchecked ROS present in aging cells can cause considerable damage by oxidizing proteins, inducing DNA mutations, mitochondrial dysfunction and lipid peroxidation [2, 3]. From these facts arose the idea that K^+^ channels, which are essential to excitable and non-excitable cells, could provide a common target for ROS [2, 4]. In fact it had been known for a long time that K^+^ channels are implicated in aging and neurodegenerative conditions characterized by high levels of ROS such as Alzheimer’s and Parkinson’s disease [5-13]. Moreover, many types of K^+^ channels including voltage-gated (Kv) [14-25], 2-P domains [26], calcium-activated (K_Ca_) [27] and G-protein coupled inwardly rectifying (GIRK) [28] K^+^ channels can be modified by oxidizing agents *in vivo* and *in vitro*. However, until a few years ago, the physiological significance of the interactions of ROS with K^+^ channels was unresolved, with the exception of a few studies which had suggested a potential role for oxidation of K^+^ channels in neuronal hypoxia [22, 29]. The breakthrough came recently, when we showed that in *C. elegans*, not only K^+^ channels undergo age-dependent oxidation but as a result of this, they impair neuronal function. In this mini-review I take stock of the field and report on the progresses achieved in these recent years.

## Oxidation of voltage-gated K^+^ channels: a common tread from invertebrates to mammals

KVS-1 (potassium voltage-sensitive subunit 1) is a voltage-gated K^+^ channel that operates in the nervous system of *C. elegans* [30]. Wild type KVS-1 currents exhibit rapid activation-inactivation and as such can be described as A-type; however, their inactivation kinetics are slower than typical A-type kinetics due to the presence of the N-inactivation regulatory domain (NIRD) which hinders the inactivation ball [31]. Most importantly, KVS-1 inactivation is redox-dependent. Generic oxidants such as chloramine-T (CHT) or hydrogen peroxide (H_2_O_2_) turn the KVS-1 current into non-inactivating, delayed rectifier type by modifying a cysteine in the N-terminus (cys113) [32]. The simple redox-dependence of KVS-1, along with the fact that *C. elegans* is genetically tractable and that the behavior mediated by the neurons where KVS-1 operates can be experimentally assessed, allowed us to study the effects of oxidation of the channel by ROS in aging worms [32]. By constructing a transgenic animal expressing a KVS-1 redox-insensitive variant (C113S), we showed that not only KVS-1 is subject to a natural process of oxidation during aging but most importantly, that this process affects behavior [32]. While our findings have provided the first experimental evidence that oxidation of a K^+^ channel by ROS is a mechanism of aging, they have also raised the question as to whether this process affects higher organisms. KVS-1 has a mammalian homolog, the KCNB1 K^+^ channel (commonly known as Kv2.1). KCNB1 is expressed in the pancreas and in the brain—mainly in hippocampus and cortex—and knock out studies have shown that the protein is important for the function of both organs [33-42]. Like its *C. elegans* homolog, also KCNB1 is directly susceptible to oxidation, even though in a more complex fashion [43]. When KCNB1 channels are exposed to CHT or H_2_O_2_ they form oligomers held together by disulfide bridges between cys73—the equivalent of cys113 in KVS-1—and other cysteines [43, 44]. KCNB1 oligomers are detected in the brains of old mice, in amounts that increase with age. Moreover KCNB1 oligomerization is exacerbated in the brain of the 3x-Tg-AD mouse model of Alzheimer’s disease [43, 45]—a brain subjected to high oxidative stress. Oligomerized KCNB1 channels do not conduct current *in vitro* [43] and probably *in vivo* [46] and under acute oxidative insults they induce apoptosis [44]. However, oligomerization is only one mechanism by which KCNB1 promotes cell death (reviewed in [47]); other mechanisms have also been amply demonstrated and will not be discussed here [48-52]. Suffice to say that KCNB1 channels exhibit multiple apoptotic profiles. In summary, the evidence at hand would suggest that moderate levels of oxidized KCNB1 channels affect hippocampal and cortical excitability, and might lead to spatial learning and memory impairment experienced during normal aging. When ROS levels further increase, such as in AD, oxidation of KCNB1 may become exacerbated and promote neuronal apoptosis.

### Oxidation of Ca2^+^-activated K^+^ channels in the brain

Another family of K^+^ channels, the calcium-activated K^+^ (K_Ca_) channels, are implicated in the aging process of the brain [53-61]. These channels have a role in the regulation of a number of physiological functions including neuronal excitability, circadian rhythm, smooth muscle tone, vasodilation of the microvasculature, K^+^ flux across endothelial cells and cell proliferation (reviewed in [62]). K_Ca_ channels exhibit a complex redox-dependence. A number of their methionine and cysteine residues can be oxidized, leading to changes in both the permeation and gating properties of these channels [27, 63, 64]. Moreover, K_Ca_ channels are endowed by accessory subunits, which further act to modulate the susceptibility of the channels to ROS [65]. Given the strong redox-dependence of K_Ca_ channels, it is not coincidental that there is growing evidence indicating that their activity increases with advancing age—probably as a result of oxidation—in a variety of organs including the brain, [53, 54], the skeletal muscle [66, 67] and the vasculature [68, 69]. Thus, studies at the single-channel level have shown that in the pyramidal neurons of the hippocampus, oxidants act to enhance K_Ca_ activity via an increase in the open probability [55]. K_Ca_ conductances are elevated in neurons subjected to high oxidative stress, such as in the brain of the TgCRND8 mouse model of amylopathy—thus of Alzheimer’s disease—as well as in cerebral ischemia [56, 57]. Finally, hyperactive K_Ca_ channels at the synapses of hippocampal and other neuron types [58-61], are the likely culprits for the altered contents of K^+^ detected in synaptosomes isolated from the brains of old mice [70]. In fact, this and other age-dependent changes in synaptosomal parameters can be prevented by antioxidant treatments. In the examples discussed above, the function of K_Ca_ channels is altered in consequence of aging. However K_Ca_ channels may also directly impact the aging process. According to a recent report, the opening of K_Ca_ channels in the inner membrane of brain mitochondria acts to decrease ROS production via respiratory chain complex 1 [71]. It is tempting to speculate that during aging the anti-oxidant action of these channels may diminish, leading to increased oxidative stress in neurons.

In summary, it appears that K^+^ channels are naturally subjected to oxidation in aging nervous systems. It remains to be demonstrated whether there is a causative relationship between these oxidative processes and behavioral and functional impairment in vertebrates. This is however likely to be the case considering that in other tissues, such as microvasculature, oxidation of K_Ca_ channels has a functional impact on physiology.

### Oxidation of K_Ca_ channels in the vasculature

Reduced blood flow is a hallmark of vascular aging. Ion channels are among the culprits for causing this deficit because they control electrical conduction which underlies vasoconstriction. One class of proteins known to undergo modification in aging vascular tissue are K_Ca_ channels [72-74]. Recently, Behringer and colleagues [68] showed that in aging mice, oxidation of small and intermediate conductance K_Ca_ channels by ROS is a major causative factor of reduced blood flow. In superior epigastric arteries of old animals, electrical conduction was impaired due to oxidation-induced hyperactivation of K_Ca_ channels. This was demonstrated by the fact that application of H_2_O_2_ reduced conduction in young animals whereas the use of scavengers, restored conduction in old arteries. More recently, Feher and colleagues [69] reported that in coronary arterioles dissected from patients undergoing cardiac surgery, conducted dilation declined with age. Like in mice, also in human vessels small and intermediate K_Ca_ conductances turned out to be hyperactivated. Thus, it appears that reduced blood flow, caused by oxidation of K_Ca_ channels by ROS, may represent a general mechanism of aging in the microvasculature.

### Conclusions

Experimental support for the notion that oxidation of K^+^ channels by ROS is a mechanism of aging is growing. While it is well-established that these proteins undergo age-dependent oxidation, it remains to be defined whether their modifications impact and if they do, to which extent, the function of the organs and tissues where they operate. Approaches based on the use of transgenic animals, like it was done with KVS-1 in *C. elegans*, will enable us to answer this question in the near future.
